# Sports and Children with Hemophilia: Current Trends

**DOI:** 10.3390/children8111064

**Published:** 2021-11-19

**Authors:** Lorenzo Moretti, Davide Bizzoca, Claudio Buono, Teresa Ladogana, Federica Albano, Biagio Moretti

**Affiliations:** 1 Orthopaedics Unit, Department of Basic Medical Science, Neuroscience and Sensory Organs, School of Medicine, University of Bari “Aldo Moro”, AOU Consorziale Policlinico, 70124 Bari, Italy; lorenzo.moretti@libero.it (L.M.); claudio.buono@uniba.it (C.B.); teresa.ladogana@uniba.it (T.L.); federica.albano@uniba.it (F.A.); biagio.moretti@uniba.it (B.M.); 2 PhD Course in Public Health, Clinical Medicine and Oncology, University of Bari “Aldo Moro”, Piazza Giulio Cesare 11, 70124 Bari, Italy

**Keywords:** hemophilia, children, sport, prophylaxis, high-impact sports, physical activity, psychological wellness

## Abstract

Hemophilia is a sex-linked recessive disorder characterized by a lack of blood factors necessary for clotting. This review aims to investigate the benefits of sports activities in children with hemophilia in terms of both physical and psychological wellness. Sports activity is necessary for children with hemophilia to preserve joints’ range of motion, reduce joint bleeding, improve muscle mass and strength, enhance proprioception and prevent secondary chronic diseases. In the past, high-impact sports were usually forbidden in children with hemophilia because of their high bleeding risk. Recent studies, however, have shown that prophylaxis therapy can allow a hemophilic child to take part in vigorous activities or high-impact sports. The benefits of sports activity in children with hemophilia are expressed by a better muscular trophism and an improved bone mineral density. Moreover, physical activity has a positive impact on children’s psychosocial well-being. Due to prophylaxis therapy, the quality of life of children with hemophilia is similar to their peers, and this has allowed an improvement in sports participation, including team sports.

## 1. Introduction

Hemophilia is a sex-linked recessive disorder characterized by a lack of blood factors necessary for clotting [1].

This disease mainly occurs in males and the deficit may be in factor VIII (hemophilia type A or classic type) or factor IX (type B) [2]. Patients with severe plasma protein deficit can have recurrent muscular and especially joint bleeding episodes, which may lead to musculoskeletal pain and physical and functional ability reduction, thus finally compromising their quality of life [3].

Consequently, it is reported that hemophilic children tend to be more sedentary compared with non-hemophilic peers because of the difficulties they may experience during physical activity [4].

This review aims to investigate the benefits of sports activities in children with hemophilia in terms of both physical and psychological wellness.

## 2. Materials and Methods

The first step consisted of a scoping literature search performed by three reviewers, CB, TL and FA, supervised by DB, using the PubMed database to select an initial pool of potentially relevant papers, originally designed to investigate the feasibility of physical activity in children with hemophilia.

The search strategy included the following terms: ((hemophilia [MeSH Terms] OR “hemophilic patient” [All Fields]) OR (hemophilic child [MeSH Terms] OR “children with hemophilia“ [All Fields])) AND (“sport” [MeSH Terms] OR (sport [All Fields]) OR “physical activity”).

The second step consisted of revising the literature review to identify papers dealing with physical activity in children with hemophilia.

Inclusion criteria were: human studies in which the authors considered the role of sports activity in children affected by hemophilia; English language; studies about children with hemophilia.

A total of 42 articles [1,3,4,5,6,7,8,9,10,11,12,13,14,15,16,17,18,19,20,21,22,23,24,25,26,27,28,29,30,31,32,33,34,35,36,37,38,39,40,41,42,43] were finally included in the present review.

## 3. Hemophilia and Sports Participation

Sports activity is necessary for children with hemophilia to preserve joints’ range of motion, reduce joint bleeding, improve muscle mass and strength, enhance proprioception and prevent secondary chronic diseases (i.e., cardiovascular disease, diabetes, cancer) [44]. To prevent joint and muscle bleeding, parents put their children with hemophilia through various exercise programs [5]. Muscle atrophy, instability and restriction of motion are the first visible signs of sedentarism [6], whereas early subclinical symptoms such as tender ligaments are found even in clinically healthy young people [1]. This leads to a lack of physical activity and exercise that results in a poor physical condition with diminished muscle strength, aerobic/anaerobic power, proprioception and flexibility [7]. Furthermore, sports activity can improve bone mineral density, which is lower in children with hemophilia than in healthy peers [8]. In the past, because of bleeding risk, sports activity was discouraged in children with chronic disease [9]. However, nowadays, due to new improvements in medical treatment, the participation of children with hemophilia in sport has improved [44].

However, even if an increase in participation in sports has been observed in children with hemophilia, aerobic activity is less practiced. This phenomenon may be explained considering that children with chronic diseases (such as cystic fibrosis or hemophilia) might have a decline in pulmonary function, which finally leads to less exercise tolerance [10]. Sports and exercise help to develop fundamental abilities, such as coordination, strength, endurance and flexibility. The muscle-to-fat ratio is improved, and, in the long term, joints are protected and bleeding episodes avoided [11].

Prophylaxis is effective to maintain a minimum level of clotting factor activity and to permit regular sports participation in children with hemophilia [12]. However, prophylaxis alone is insufficient to protect from bleeding and joint damage [13]. In fact, in children with hemophilia, it is important to maintain weight within a healthy range to prevent an overload of the joints, especially the knees and ankle [14]. Furthermore, sports exercise increases factor VIII levels and could modify coagulation parameters in mild/moderate hemophilia [15]. It is therefore reported that an increased plasmatic lactate concentration, secondary to anaerobic exercises, for instance, may affect FVIII clearance, thus improving the patient’s coagulation [1].

In the past, high-impact sports were usually prohibited in children with hemophilia due to the high risk of bleeding injuries [16]. In the 1970s, it was a common practice to discourage any type of sports because of the risk of bleeding episodes, but today, the participation in sports activities by hemophilic patients has improved, and physical activity is considered healthy for this type of patient [17] even if high-impact sports are still not recommended. Nowadays, on the other hand, different guidelines are available to regulate hemophilic patients’ sports participation; hemophilia type and severity play a key role in the correct sports activity choice [18,19]. According to some hemophilia centers, the choice of activities should reflect individual basis such as: preference/interest, ability, physical condition and resources [7]. Participation in non-contact sports (swimming, running and walking) should always be promoted, but high-impact sports (rugby, boxing, football and basketball) or sports such as motocross (endowed with a higher injury risk) are often discouraged even on good prophylactic therapy [7,11].

In the United States, the National Hemophilia Foundation (NHF) proposes the stratification of activities into safe, safe-to-moderate, moderate, moderate-to-dangerous and dangerous risk groups. The safe through moderate categories can be routinely recommended with the proper preparation [20]. Another stratification in high-impact and low-impact sport was proposed by Ross and Goldenberg in 2009: high-impact sports include soccer, basketball, baseball, bowling, gymnastics, field hockey, running, skiing, snowboarding, soccer, softball, tennis and track and field, while low-impact activities include weight training, cycling, Frisbee, golf, swimming and walking/hiking [21].

However, is it right to forbid children with hemophilia to participate in high-impact sports even if they are on prophylactic treatment?

According to some authors, prophylactic therapy can allow a hemophilic child to engage in vigorous activities or high-impact sports [44]. An article by Ross et al. [21] showed that children with hemophilia on prophylaxis could participate without any increased risk of joint bleedings.

The American Academy of Pediatrics (AAP) Committee on Sports Medicine and Fitness has divided childhood activities according to risks and formulated guidelines for sports participation [22]. The AAP has recommended that children should engage in trampoline activities only in professionally supervised settings due to the high risk of fractures, hospitalization and risk of bruises and other injuries [23]. For the same reason, no children should participate in boxing because this activity encourages injuries especially to the head and neck [24]. Additionally, the dangers of concussion related to US football and soccer have recently received attention, with recommendations for carefully monitoring children after an event [25]. Nonetheless, the AAP recommends participation in sports activities for children with bleeding disorders [21].

In 2017, the National Hemophilia Foundation (NHF) proposed some guidelines for athletic participation by patients with a bleeding disorder [20]. Therefore, a minimum of 60 min of exercise per day, with appropriate supervision, is recommended for children after receiving prophylaxis.

## 4. Treatment of Sports Injuries in Children with Hemophilia

Significant bleeding episodes in hemophilic patients are typically treated with the administration of missing clotting factors (factor VIII or IX), whereas they could be managed by bypassing agents or antifibrinolytic medication [1]. Missing factors should be administrated to permit regular sports activities in children with hemophilia with a severe deficiency (when the factors activity is lower than 10–20%) [27]. The high adherence in young children is related to the benefits of sports activity also without parents’ supervision [26,45,46,47,48,49]. Non-adherence to prophylaxis could be responsible for an increase in joint bleeding, reduced quality of life and absence from school. Children should receive regular infusions to reduce the risk of bleeding to preserve joint wellness [28]. An alternative treatment, in the case of minor bleeding episodes, is the use of desmopressin (intravenously or intranasally) [1]. A study published in 1980 showed that desmopressin also increases factor VII plasma concentrations through the release of VWF (Von Willebrand Factor) [29]. The main complication after treatment with clotting factor concentrates is the development of inhibiting antibodies directed against some parts of factor VIII/IX, and these are the cause of a reduction in its coagulant activity [30]. Usually, inhibitors are produced in children within the first 50 days of treatment and they are the cause of an increase in the risk of bleeding episodes [31]. In the past, the usage of plasma, containing clotting factors, from unscreened donors made the transmission of blood viruses easier (HBV, HCV and HIV). Nowadays, donors are tested before blood donation [1].

## 5. Bleeding Prevention in Hemophilic Children

In children with lower (5% or less) factor levels, a higher bleeding risk has been observed during sports activity. It is reported that an increase of 1% in the factor level with treatment before sport correlates with a decreased bleeding risk by 2% [32,38,39,40,41,42,43]. Assessments of joint and muscle function before sport selection in children with hemophilia are required [33]. In addition, they require a complete evaluation, which should include: an analysis of balance and coordination, aerobic capacity and body fat content [23]. Although the risk of injury cannot be eliminated, protective measures can be taken to reduce the risk of injury: the use of helmets, facemasks, shin guards, kneepads, wrist and forearm guards according to the type of sports activity [34]. The risk of serious bleeding and the number of hemorrhages can be radically decreased with the use of prophylaxis with factor VIII and IX concentrates [21].

A higher factor level at the time of injury is a predictive factor of bleeding events. These observations offer the opportunity to minimize bleeding risks during participation in sports [35,50,51,52,53]. A way to reduce the risk of bleeding is to divide the dose of the prophylactic factor by the number of days per week and concerning sports participation [23]. In such a way, the factor level at the time of collision may be increased, reducing the risk of bleeding episodes [12,25,54,55,56].

Newer longer-acting clotting factors may improve the maintenance of a factor level enough to prevent bleeding. In addition, strengthening and warming up before sports participation may reduce the rate of sports injuries. The risk of participation in collision sports is only moderately increased in hemophilic boys in prophylactic therapy, so the risk of sports injuries in hemophilia becomes similar to that of their healthy peers [36,37].

## 6. Psychosocial Well-Being and Sports Activity

The positive impact of sports activity on psychosocial well-being is well known, and some studies have recently investigated the relationship between physical activity and the psychosocial dimension in hemophilic patients.

Von Macksen et al. [57], in a multicenter, cross-sectional study, have recently described the impact of sport on health-related quality of life (HRQoL), physical performance and clinical outcomes in adult patients affected by hemophilia. The authors recruited fifty hemophilic patients with mild (*n* = 12), moderate (*n* = 10) or severe (*n* = 28) hemophilia A (70%) or B (30%). Among the recruited patients, 36% of participants reported not participating in any sport, mainly because of their physical condition, whereas the remaining 64% of participants reported undertaking sporting activity, including high-impact sports. The authors showed that patients participating in more sport reported significantly better HRQoL than those participating in less sport (*p* < 0.005).

Similar findings were reported by Sondermann et al. [47] in hemophilic children. These authors showed that the increase in physical activity did not correlate with an increase in bleeding events in the recruited children. Moreover, a positive impact on the children’s quality of life and participation in social/school activities was observed.

## 7. Conclusions

The benefits of sports activity in children with hemophilia are expressed by a better muscular trophism and an improved bone mineral density. Moreover, physical activity has a positive impact on children’s psychosocial well-being.

Due to prophylaxis therapy, the quality of life of children with hemophilia is similar to their peers and this has allowed an improvement in sports participation, including team sports. While in the past, due to the high risk of injuries, participation especially in team sports had been discouraged, nowadays sports activity has been promoted to achieve better physical and social wellness.

## Data Availability

Not applicable.
